# Effect of Elevated Temperature on the Bond Strength of Prestressing Reinforcement in UHPC

**DOI:** 10.3390/ma13214990

**Published:** 2020-11-05

**Authors:** Petr Pokorný, Jiří Kolísko, David Čítek, Michaela Kostelecká

**Affiliations:** Klokner Institute, Czech Technical University in Prague, 166 08 Prague, Czech Republic; Jiri.Kolisko@cvut.cz (J.K.); David.Citek@cvut.cz (D.Č.); Michaela.Kostelecka@cvut.cz (M.K.)

**Keywords:** ultra-high performance concrete (UHPC), prestressing reinforcement, bond strength, elevated temperature, pore size distribution, brass coated fiber

## Abstract

The study explores the effect of elevated temperatures on the bond strength between prestressing reinforcement and ultra-high performance concrete (UHPC). Laboratory investigations reveal that the changes in bond strength correspond well with the changes in compressive strength of UHPC and their correlation can be mathematically described. Exposition of specimens to temperatures up to 200 °C does not reduce bond strength as a negative effect of increasing temperature is outweighed by the positive effect of thermal increase on the reactivity of silica fume in UHPC mixture. Above 200 °C, bond strength significantly reduces; for instance, a decrease by about 70% is observed at 800 °C. The decreases in compressive and bond strengths for temperatures above 400 °C are related to the changes of phase composition of UHPC matrix (as revealed by X-ray powder diffraction) and the changes in microstructure including the increase of porosity (verified by mercury intrusion porosimetry and observation of confocal microscopy) and development cracks detected by scanning electron microscopy. Future research should investigate the effect of relaxation of prestressing reinforcement with increasing temperature on bond strength reduction by numerical modelling.

## 1. Introduction

UHPC (ultra-high performance concrete) is a relatively new (1990s) composite construction material of cement base. Compared to conventional concretes, the UHPC shows improved mechanical properties, i.e., compressive strength, abrasive resistance, and durability. Cement composite of UHPC grade is commonly classified as a standardized material with strength above 150 MPa [1], potentially reaching up to 810 MPa [2] with optimal composition. Improvement of mechanical properties compared to the common grade of concretes is achieved by decreased porosity and increased homogeneity of the material (inhibiting the number of cracks on the phase interfacial transition zone of fine aggregate and binder). This is why high-strength grades need to have different compositions [1,3]. Such compositions are characteristic for their high amount of cement and the addition of compounds with latent hydraulicity (silica fume, granulated blast furnace slag, fly ash, etc.) and very fine aggregates (natural or refined quartz sand with grain diameter smaller than 600 μm) and fibers (commonly base, fibers from various polymers, glass, wood, and many others are also feasible) [1,3,4,5].

**Figure 1:**
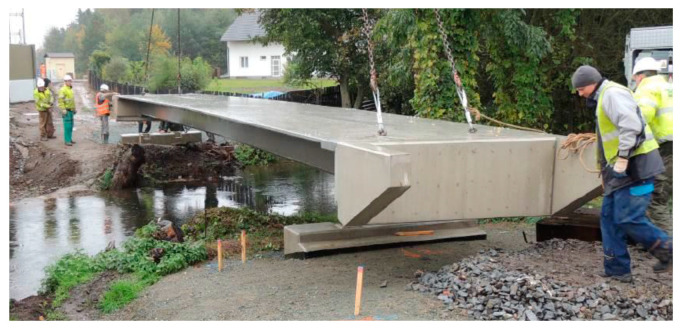
Installation of the prestressed ultra-high performance concrete (UHPC) girder of a beam footbridge (2015—village Ceperka across Opatovicky canal, Czech Republic).

**Figure 2:**
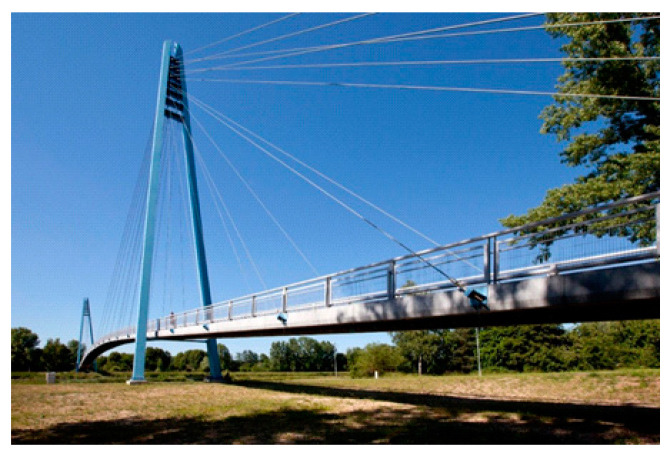
Footbridge from UHPC in Celakovice (2014—across the Elbe river, Czech Republic).

Current application of concretes of the highest grade in civil engineering is still relatively rare, with few successful applications as bridge decks, industrial floors, building facades (for example, Museum of Civilizations of Europe and the Mediterranean), wind turbine towers or prefabricated prestressed girders [4,6,7]. Other interesting applications of ultra-high performance concrete are lightweight shell structures (especially roof construction—for instance, the light rail roof platforms in Calgary) and permanent formwork (the advantage is the acceleration of the technological process of building construction). Use of UHPC for prefabricated prestressed girders of long-span bridges or footbridges (Figure 1 and Figure 2 show the applications in the Czech Republic) seem to be very promising since the material has high resistance against the environment (de-icing salts, SO_2_, and freezing resistance) and is a light-weight material [8,9]. Upgrades are a brand new field for the application of UHPC. High strengths of ultra-high performance concrete are particularly advantageous for seismic upgrading of existing structures [6,9].

For the use of constructional components made of UHPC, especially prestressed girders, the resistance against high temperature is also of importance (natural or accidental fires). Unlike conventional concretes (NSC—normal strength concrete), the UHPC shows higher sensitivity when exposed to high temperatures for longer periods. With increasing temperature, UHPC concrete shows a decrease of its advantages and even thermally induced explosive spalling may occur [10,11,12,13]. Reasons are lower porosity, lower connectivity of pores, dense microstructure of UHPC compared to NSC, and higher sensitivity to elevated thermal stress and elevated vapor pressure of free or bound water in the cementitious matrix [11,14]. The rate of pressure increase inside composite materials can result in a rapid loss of material integrity. If thermally induced explosive spalling, typical for UHPC, does not occur, the decrease of mechanical properties (compressive strength, flexural strength, etc.) is caused by an increased water evaporation, increasing the porosity of compacted materials, coarsening the pore structure and decomposing hydration products (predominantly C-S-H gel and CH—portlandite) [15].

There are many research studies evaluating the dependance of the composition and microstructure of NSC/UHPC composites on their mechanical properties (overview in, e.g., [11]). Prestressed UHPC components are necessary to monitor from the perspective of bond strength in the case of exposure to high temperatures (structural surface exposed to fire). Reduced bond strength of prestressed reinforcement can have a negative effect on the load-bearing capacity of the structure even before the temperature starts affecting the mechanical properties directly [16].

Similar to ribbed reinforcement, the bond strength of prestressing reinforcement with concrete (for prestressed components) is given by the mechanical properties of the concrete (surface roughness of the reinforcement, anchoring length, temperature during loading and other parameters). Reduction of mechanical properties of UHPC due to increasing temperature will have a negative effect on the bond strength of any reinforcement in general [17].

It is recognized that UHPC structures can be more vulnerable to fire and elevated temperatures. The use of polypropylene (PP) fibers may mitigate this unfavourable effect. Various studies showed that the addition of about 0.5% of polypropylene fibers improved the fire resistant properties of UHPC (mainly preventing thermally induced explosive spalling). Melting of the polypropylene fibers (starting above 170 °C, most of PP fibers being completely melted at 600 °C) creates large pores and allows for releasing the internal pressure. However, cracks may develop at surface [18,19,20,21]. Other research works attribute a positive effect of thermal mismatch between embedded fibers and matrix holds the key in obtaining an interconnected network of cracks in the matrix [22,23].

A partially improved behaviour at elevated temperatures was observed with the addition of steel fibers in UHPC matrix. The presence of steel fibers in UHPC samples improves energy absorption and reduces cracking [18,24,25].

The current literature does not present sufficient relevant data on the bond strength between a prestressing reinforcement and a UHPC depending on temperature. This paper describes these phenomena in relation to changes of the mechanical properties of UHPC (compressive strength, abrasive resistance, durability). Regarding to the mechanical properties of UHPC, changes in porosity at the concrete/steel fiber phase interfacial transition zone (scanning electron microscopy) evaluated in relation to increasing temperature. Changes in the phase composition of UHPC samples were also measured using X-ray powder diffraction.

## 2. Materials and Methods

Concrete samples (cubes of UHPC) were produced using a mixture of CEM II/A-S 52.5 N, the exact composition is shown in Table 1. Individual UHPC samples were produced using the mixture recipe shown in Table 2. The mixture also contained brass-coated steel fibers (total content of 1.5 vol.%). The chosen value of water coefficient of 0.25 (*w*/*c*) is common for high-strength concretes. The mixing was done in a standard mixer device. After deflocculation of the mixture, the brass coated steel fibers were added. All components were then mixed for 5 min to homogenize the structure (not aligned brass-coated steel fibers). This UHPC mixture is self-compacting.

**Figure 3:**
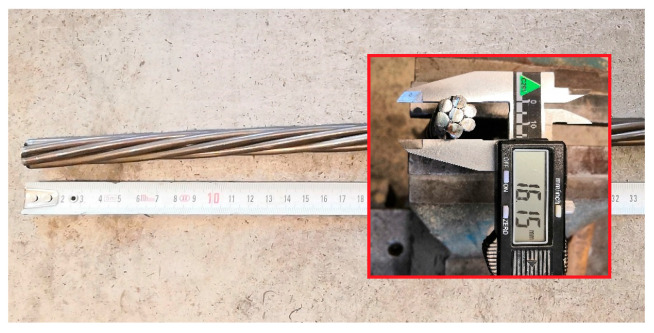
Visual characterization of non-prestressed tendon used for the measurement of the bond strength in UHPC.

According to CSN 73 1333 [26] and RILEM RPC 6 [27], the bond of prestressing reinforcement in concrete is tested for non-prestressed wires or tendons. This procedure is adopted here to test the bond of prestressing reinforcement in UHPC at elevated temperatures. The recommendations of ASTM E 119 for control of thermal exposure (temperatures in furnace and on the surface of a specimen) during mechanical tests (bond test in this study) and protection and conditioning of specimens are taken into account [28]. Parameters of prestressing reinforcement are listed in Table 3. Figure 3 shows the prestressing reinforcement under investigation.

**Figure 4:**
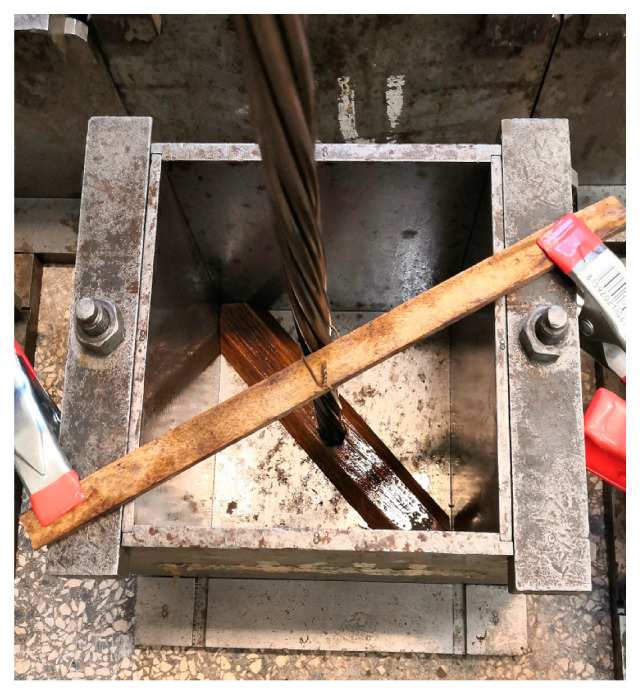
Anchoring of prestressing reinforcement in the axis of the cube according to the standard [26].

UHPC samples (cubes with an edge length of 150 mm—the same cube samples were performed for compressive strength measurements) for bond strength tests were fabricated according to the standards [26,27], including anchoring of prestressing reinforcement in the axis. Storage does not require the separation (PVC tube) of the prestressing reinforcement from the UHPC matrix [26,27]. Correct anchoring placement was achieved by using a trapezoid wooden insert on the bottom of the mold and squared timber on the top (Figure 4).

**Figure 5:**
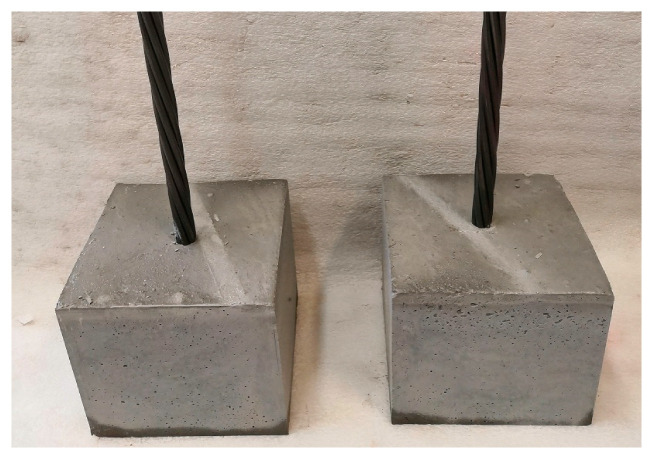
Overview of two UHPC samples for bond strength tests.

Individual UHPC samples for bond strength and compressive strength tests were cured the first day after casting in dry atmosphere (65% RH, 20.5 ± 1 °C) and then in distilled water for the remaining 28 days. After curing (Figure 5), the bond strength and compressive strength tests were performed immediately. Ten sample cubes from each group of samples (for a single test temperature) were fabricated for compressive strength and bond strength tests.

For all laboratory experiments, specimens of UHPC are pre-dried at 105 °C to evaporate the capillary moisture (to avoid thermally induced explosive spalling). Total pre-dried time was set at 48 h.

**Figure 6:**
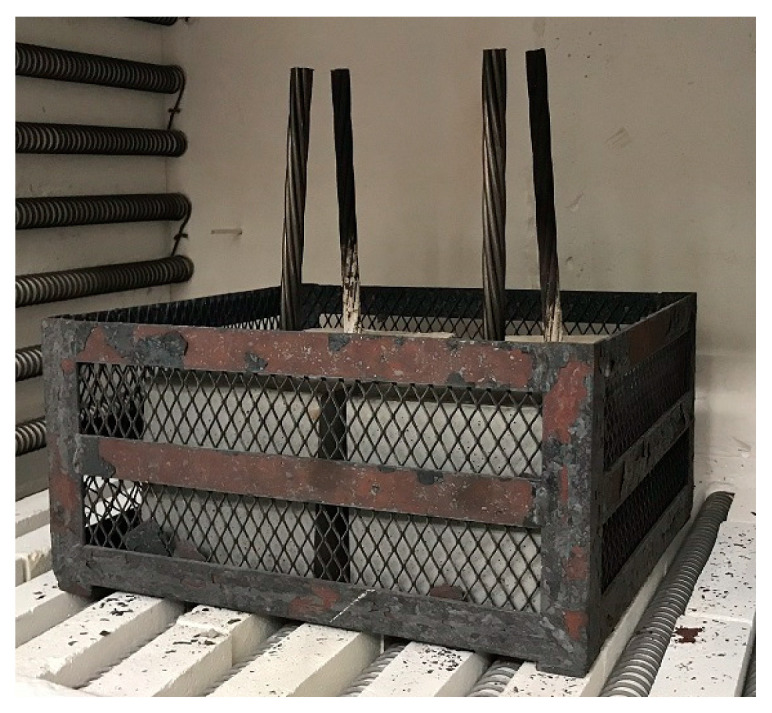
Example of the UHPC samples with prestressing reinforcement in the furnace.

Individual UHPC specimens (with and without prestressing reinforcement) were placed in a furnace (Figure 6). The specimens were heated gradually, with the initial rate of 1 K/min and held after reaching the target temperature for 1 h. Colling was done in a laboratory atmosphere.

**Figure 7:**
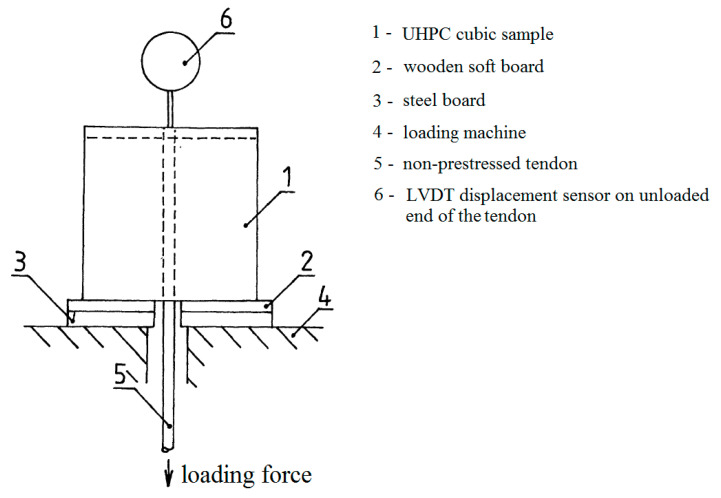
Experimental setup of a pull-out test of the bond strength between the prestressing reinforcement (non-prestressed tendon) and concrete.

**Figure 8:**
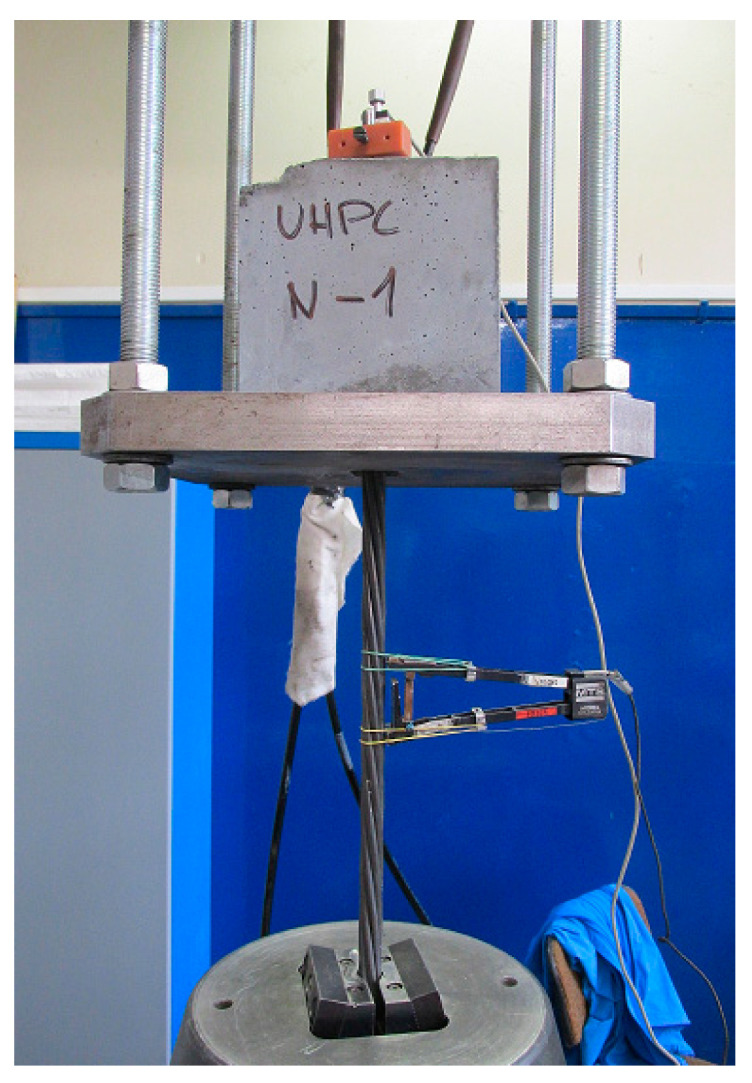
Experimental setup for bond strength (pull-out) test according to [26,27,29].

The testing of the prestressing reinforcement with UHPC was done by recording the increasing bond stress slip on the unloaded end of the reinforcement (loading machine MTS 500 kN by using MTS FlexTest, Eden Prairie, MN, USA). The test was controlled by the displacement of the unloaded end of the reinforcement (a stable loading rate of 0.005 mm·s^−1^)—see the pull-out test experimental setup in Figure 7 (reprinted and remarked from [26,27]). The loading was terminated after reaching a slip of 2.0 mm. The experiment setup before the test is shown in Figure 8.

After the compressive strength and bond strength tests, the concrete samples were studied in detail. The UHPC samples were cut approximately through their center (yet outside the UHPC/reinforcement phase interfacial transition zone). On these samples, mercury intrusion porosimetry, SEM, and confocal observations were performed. In the case of phase composition alteration (X-ray powder diffraction—PANanalytical X’Pert3 Powder (Malvern, UK) using CuKα radiation over the angular range of 5–90°, semi-quantitative analysis was performed using RIR (reference intensity ratio) values from the PDF4 + database of the HighScore Plus software, 1.0, Malvern, UK), the samples were mechanically homogenized with the goal of acquiring powder samples. The coarsest fraction (silica sand) was removed by sieving, followed by the removal of brass-coated fibers (using a manual permanent magnet).

For mercury intrusion porosimetry (AutoPore IV - Micromeritics, (Norcross, GA, USA) measurements in the setup for concrete porosivity), a total of 7 specimens from each exposure set were prepared in the shape of a cube with a 2 cm edge; prepared by grinding in ethanol. For confocal microscopy, a surface of about 5 cm^2^ was scanned (for the actual measurement of one group of samples, 10 samples were always prepared, each with a specific surface area of approximately 0.5 cm^2^). SEM microscopy for the matrix and ITZ—aggregate/matrix, brass-coated steel fiber/matrix analysis included two samples from each exposure set (cube samples with a measured surface about 4 cm^2^). The samples were also deliberately selected with the possibility of imaging the aggregate/matrix and brass-coated fibers/matrix.

## 3. Results and Discussion

The following section summarizes the results of the compressive strength tests of UHPC, the tests of the bond strength between prestressing reinforcement and UHPC, the X-ray powder diffraction, mercury intrusion porosimetry, image analysis by confocal microscopy, and scanning electron microscopy of UHPC.

### 3.1. Compressive Strength

**Figure 9:**
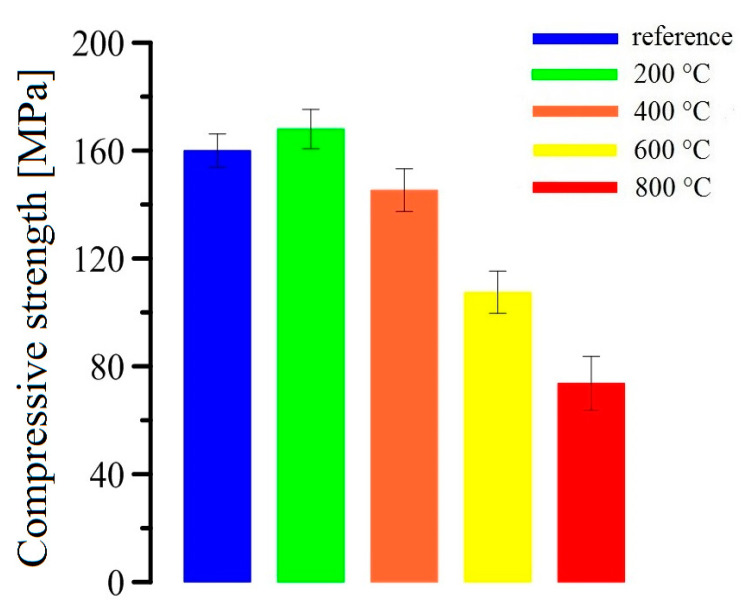
Compressive strength of UHPC specimens after their exposure to elevated temperatures.

The results of measurement of UHPC specimens are shown in Figure 9. The average value of compressive strength (10 measurements for one group of samples) for a reference sample (exposure at 20 °C) is depicted by the blue bar and for the samples exposed at 800 °C by the red bar. At 200 °C (green bar), a minor increase of compressive strength for the UHPC samples was detected (approximately 10%), which is in accordance with the previously published results for an UHPC of similar composition [30,31,32]. The decrease of bond strength of UHPC samples with increasing temperature can be explained by changes in composition (dehydration and, at high temperatures, decomposition of hydration phases) and microstructural changes (porosity increase, cracks on the phase interfacial transition zone of aggregate/matrix and/or fiber/matrix) [9,11,14]. The increase of compressive strength in the samples exposed at 200 °C is caused by the promotion of cement hydration process and pozzolanic reaction, resulting in a denser microstructure [14,33]. The reactivity level of silica fume can be increased from 10% to 75% by growing the temperature from 90°C to 250 °C [14,34]. Some research works describe an increase of compressive strength in UHPC samples up to an exposure temperature of 300 °C. Furthermore, the temperature of 300 °C is considered as the limit for the positive effect of the reactivity level of silica fume. A declining trend in mechanical properties of UHPC exposed beyond 300 °C was mainly due to the weakening of the internal microstructure [18,35]. The change of compressive strength in the temperature range 200–300 °C very closely depends on the type of added fibers; for the specimens with polypropylene fibers, a steep decline of compressive strength can be observed due to melting of the fibers (formation of a specific porous structure). In contrast, the addition of steel fibers slightly increases the compressive strength above 200 °C by about 1–5% [36,37].

### 3.2. Bond Strength

As already indicated, bond strength of prestressing reinforcement is also affected by the mechanical properties of concrete. Prestress transfer bond at the interfacial transition zone tendon/concrete according to [16,38] (*σ_bpd_* in Equation (1) below) takes into account a wide range of mechanical properties of concrete, estimated as the design concrete tensile strength (*σ_ct_*(*t*)—the average tensile strength of concrete at the time) during relaxation. Equation (1) also includes constants; *η*_*p*1_—coefficient which takes into account the type of tendon, *η*_*p*2_—factor that consider the position of the tendon during concreting.

(1) $$\sigma_{bpd}=\eta_{p1}\eta_{p2}\sigma_{ct}\left(t\right)$$

To simplify, the mechanical properties of concrete for bond strength tests can be characterized by compressive strength, both in the case of prestressing reinforcing bars [26] and conventional rib reinforcing bars [39,40]. Bond strength for concrete with an arbitrary steel profiled reinforcement (*T_c,i_*—bond force) can be characterized by the bond strength factor if (Equation (2) [41,42]). Bond strength factors (*i_f_*—Equation (2)) take into account the contributions of individual physical forces—*i_fad_* (factor of adhesion force), *i_ff_* (factor of friction force), *i_fσ_* (factor takes into account mechanical properties of concrete). Upper indexes *A* (i.e., specifically *A^b^* and *A^r^*) indicate the area of the main action of the forces, *b*—body of the tendon, *r*—ribs of the tendon (profiling of reinforcement).

(2) $$T_{c,i}\approx i_{f_{ad}}^{A^{b},A^{r}}+i_{f_{f}}^{A^{b}}+i_{f_{\sigma }}^{A^{r}}$$

(3) $$i_{f_{\sigma }}^{,A^{r}}\gg i_{f_{ad}}^{A^{b},A^{r}}+i_{f_{f}}^{A^{b}}$$

The factor $i_{f_{\sigma }}^{,A^{r}}$ takes into account the mechanical properties of the concrete, summarized simply by the compressive strength of the covering layer. In the case of profiled reinforcement (rib reinforcing bars, indented bars, prestressing reinforcing bars or wires, etc.), the factor has the largest impact on the total bond strength ($i_{f_{ad}}^{A^{b},A^{r}}$—an adhesion factor including an adhesion binder in concrete; $i_{f_{f}}^{A^{b}}$—the friction coefficient)—see Equation (3) [42].

**Figure 10:**
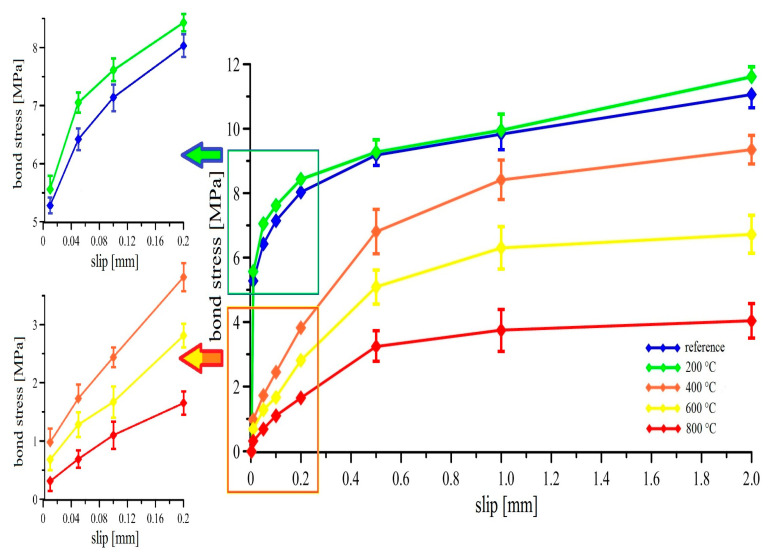
Bond strength curves of the prestressing reinforcement in the UHPC samples exposed to elevated temperatures.

There is an obvious correlation between the measured average compressive strength values and the bond strength (the average bond strengths are shown in Figure 10), which corresponds to the basic relation ((Equation (2)) between the mechanical properties of the concrete and the bond strength of prestressing reinforcement [11,14].

Similar to the compressive strength, a slight increase of the bond stress detected on the curve for the samples exposed to 200 °C in individual slip points is evident. The shape of the curves at low slip values (0.04–0.2 mm) suggests higher bond strength of the reinforcement in UHPC for the sample exposed to 200 °C compared to the reference sample. This observation is further supported by the higher slope of the bond strength curve for a slip in the interval <1.0 mm; 2.0 mm>, where the bond strength constantly increases. The highest slope for both the discussed sample sets (reference; exposed to 200 °C) occurs in the smallest slip region, specifically <0.02 mm; 0.05 mm> and is identical for both groups. The increase of the measured bond stress values for the samples exposed to 200 °C compared to the reference group can be explained by a denser microstructure of UHPC [14,33] and correlates well with the average of the compressive strength values as also observed in [39,40,43].

The decrease of the bond strength of the prestressing reinforcement in UHPC is obvious on the specimens exposed to 400 °C, 600°C, and 800 °C. The trend of the bond strength curves at 400 °C shows a 20% decrease compared to the reference group. The slope of the curve is still comparable to the reference group and to the group exposed to 200 °C. The group exposed to 600 °C shows a significant reduction of bond strength, about 45% relatively to the reference. Moreover, the bond stress increase in the slip range <0.5 mm; 2.0 mm> is only 0.5 MPa. The specimen exposed to 800°C had the bond strength reduced by approximately 70%, and the bond stress did not increase in the slip range above 0.5 mm. The reduction of bond strength in concrete with prestressing reinforcement for the group of UHPC specimens exposed to higher temperatures (400 °C; 600 °C; 800 °C) is evidently linked to the reduction of compressive strength (see Figure 9). A similar correlation between the bond strength, respective transmission length of the prestressing strands, and the compressive strength of UHPC has been described in [44,45,46] and the results further confirm these findings.

The bond strength of the prestressing reinforcement in UHPC is also closely influenced by the mechanical properties of the concrete at the interfacial transition zone; local accumulation of pores at ITZ can lead to a statistically less predictable reduction in the bond strength. In relation to this, it is necessary to verify the bond strength of the prestressing reinforcement for a statistically significant number of samples [36,37].

Regarding the results of testing the bond strength between the prestressing reinforcement and UHPC (see Figure 10), the validity of the general equation *T_c,i_* (Equation (2)) for a conventional ribbed bar reinforcement can be confirmed in the area with already significant deceleration of the bond stress growth (~0.5 mm slip) [42].

The effect of relaxation due to increasing temperature on bond is not considered in this work. The relaxation may be as indicated by the fire tests of prestressed slabs by Bailey and Ellobody [47]. They conclude that further experimental investigation of the bond behaviour at elevated temperatures, possibly affected by relaxation, is required to properly explain the observed changes in behaviour of the slabs at elevated temperatures.

### 3.3. X-ray Powder Diffraction

**Figure 11:**
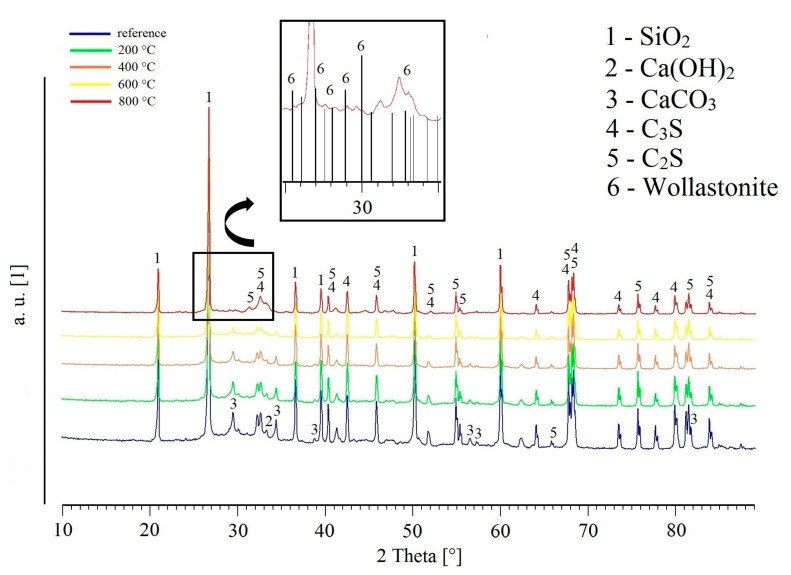
XRD patterns of UHPC samples after exposure to elevated temperatures.

Figure 11 shows the XRD patterns of UHPC powdered samples. The reduction of peak intensity characterizes the content of Ca (OH)_2_ (CH-portlandite) during exposure to 200 °C (confirmation of the silica fume pozzolanic reaction rate [3,7]). The Ca (OH)2 peak disappears completely after the exposure of UHPC to 600 °C, in agreement with other research works focusing on the decomposition of portlandite (CH) at temperatures above 600 °C [14,48,49]. CaCO_3_ (calcite) peaks are not evidently present for specimens exposed to 800 °C (the decomposition of calcite occurs at approximately 700 °C [50,51]). The decomposition of Ca (OH)_2_ and CaCO_3_ can be partially related to the decrease of mechanical properties (compressive strength and bond strength) [14]. More significant is the effect of dehydration and partial transformation of C-S-H gel (~700 to 800 °C) [52,53]. It has been shown that above 800 °C, the amorphous C-S-H gel transforms into crystalline wollastonite [54,55]. This change is also related to the decrease of the UHPC mechanical properties. The presence of wollastonite mineral (an amount of about 1–5 wt.%) was detected by XRD (see details in Figure 10). A more accurate analysis of the wollastonite content is prevented by the SiO_2_, C_3_S and C_2_S phase overlap especially in diffraction angle area 25–35°. In some research works it is considered that the faster transformation of the C-S-H gel to wollastonite occurs only at a temperature of 900 °C, when this transformation already has a fundamental effect on the development of the pore structure and thus on the compressive strength of UHPC [36,37].

It is also obvious that for the samples exposed to 400 °C, the evolution of porosity and cracks affects the compressive strength and the bond strength between the prestressing reinforcement and UHPC (the decomposition of individual phases at this temperature has not yet been proven).

### 3.4. Mercury Intrusion Porosimetry (MIP)

**Figure 12:**
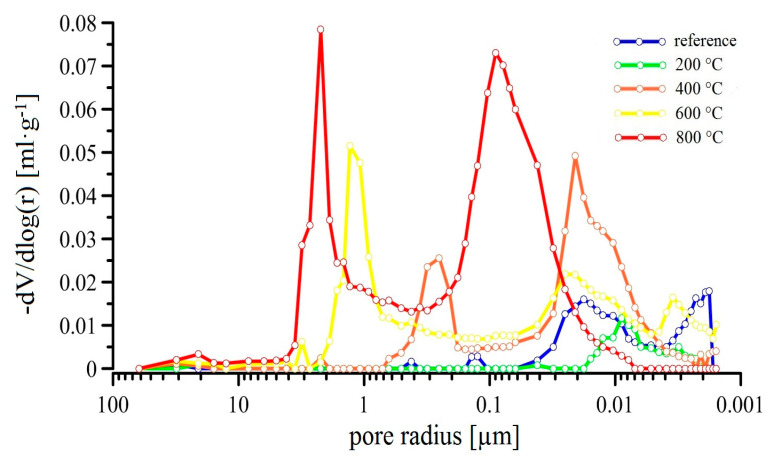
Pore size distribution of UHPC samples after their exposure to high temperatures.

Thermal loading increases the porosity of UHPC and has a coarsening effect, which in the end affects the mechanical properties [11,14,56,57,58]. An increase of porosity is initially caused by water evaporation in the interlayer and gel pores, later by the decomposition and dehydration of hydration products [7,14]. Some authors elaborated that the increase of porosity is caused by significant changes on the (ITZ) fiber/matrix phase interfacial transition zone, more specifically cracks forming as a result of an expansion of steel fibers or their thermal oxidation [14,59,60]. The results of mercury porosimetry are shown in Figure 12. Comparing the pore distribution, an UHPC specimen exposed to 200 °C shows lower porosity than the reference sample. The reason is the acceleration of the hydration processes described above (pozzolanic reaction). The increase of activity of the silica fume reaction ensures the filling of pores still saturated with water. These would be the pores with a lower diameter [3,7], in this case specifically 10–30 nm. During the exposure of UHPC to 200 °C, even very small gel pores with a diameter of several nanometers are filled. In some articles it is stated that at the initial temperature load, pores with a radius of less than 10 nm are a priori filled [35,61,62]. Although it is evident that the exposure of the samples to temperatures up to 200 °C causes a decrease in porosity. This fact was carefully confirmed, e.g., in [63]. In accordance to other authors, a significant increase of porosity occurs in the temperature range 400–800 °C [64,65]. The results suggest that the diameter of the pores is also increased with temperature. During the exposure to 400 °C the pores with diameter 10–30 mm grow, which is very likely related to the significant water evaporation from hydration products [7,11,14]. The decomposition of the hydration products is not yet significant (C-S-H gel, portlandite—CH); however, it can be ascribed to the formation of 100–500 nm pores. At temperatures 600 °C and 800 °C, a significant number of large pores are formed (several μm), resulting in thermal stress in the UHPC. The mechanism is probably a more pronounced decomposition of hydration products (compared to 400 °C) and increasing porosity at the fiber/matrix phase interfacial transition zone [7,14,66]. The increase in porosity in the UHPC samples loaded with increasing temperature (100, 200, 300, and 400 °C) was also detected by measuring water porosity. [67].

### 3.5. Image Analysis by Confocal Microscopy

**Figure 13:**
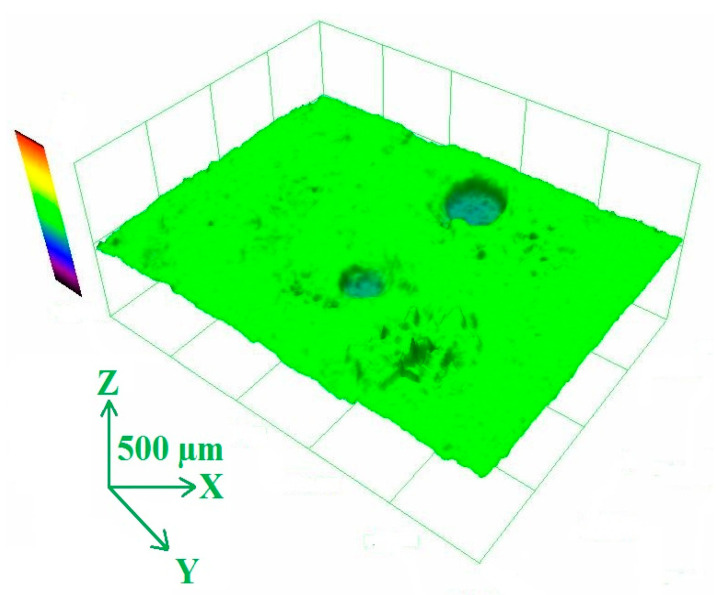
3D depth projection of the porous structure of UHPC sample after exposure to 200 °C.

**Figure 14:**
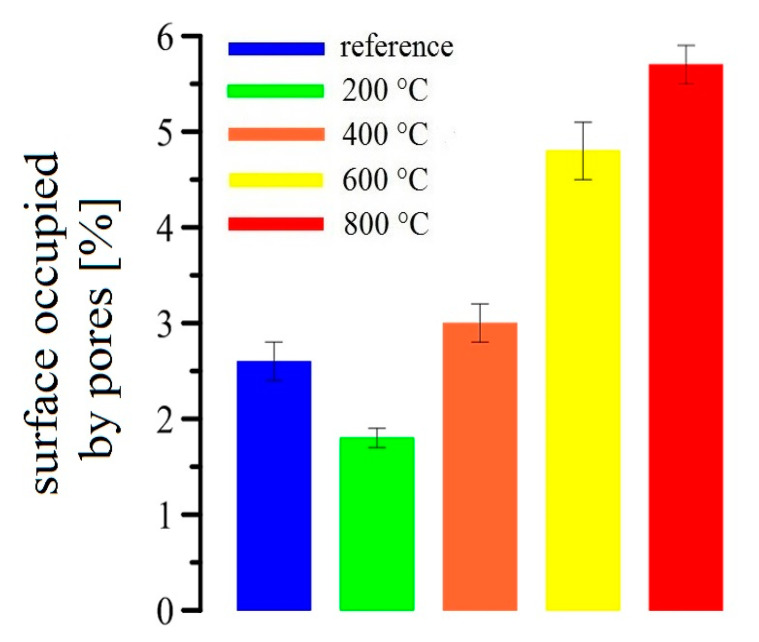
Results of image analyses of UHPC specimens after their exposure to elevated temperatures.

Changes in the pore structure were verified using image analysis (evaluation of the total surface occupied by pores), scanned by a confocal microscope. One example of a 3D depth projection of the porous structure of an UHPC sample is shown in Figure 13. The summary of the image analysis results are shown in Figure 14. The data correlates well with the mercury porosimetry (Figure 12). The lowest areas occupied by pores were found in the reference samples (~2.5%) and in the samples exposed to 200 °C (~1.5%). The surface area of pores increases with temperature.

At 600 °C, a significant increase of the pore area (almost 2%) was detected. The results are compared to the samples exposed to 400 °C. The difference between the samples exposed to 600 °C and 800 °C is not that significant (~1%). It is apparent that at 600 °C, pores of different diameters occur (amplifying degradation processes in UHPC). At 800 °C, large cracks appear on the surface of UHPC samples, which render the precise total pore area measurement impossible. It is expected that the analysis somewhat underestimates the total pore area for the samples exposed to 800 °C.

Changes of the total pore area measured in this work by confocal microscopy confirm the general trends described elsewhere [7,14,65].

Some articles describe that due to the thermal interaction (even at relatively low temperatures) between the matrix/steel base fibers, radial cracks of unit length up to tens of μm are formed, which have a very negligible volume, but already have a non-negligible surface [37,68]. Due to their small volume, these pores/cracks are not detectable by mercury intrusion porosimetry. Moreover, from this point of view, the results provided by confocal microscopy are more objective than the results provided by mercury intrusion porosimetry (MIP).

A small increase of porosity of UHPC specimens exposed to 400 °C (by about 0.5% relative to the reference) causes a 20% reduction of the bond strength to the prestressing reinforcement (see above).

### 3.6. Scanning Electron Microscopy (SEM)

After high-temperature exposure, integrity (morphology) was studied using scanning electron microscopy. Both the matrix of the UHPC samples and the phase interfacial transition zone between the matrix/brass-coated steel fibers and the matrix/aggregate were studied. Cross sections were prepared from the sites with high aggregate concentrations near the brass-coated fibers. The degradation degree of the coating (brass) and the surface of the steel fibers (partially melt) was verified using elemental maps (Fe and Zn, eventually O).

**Figure 15:**
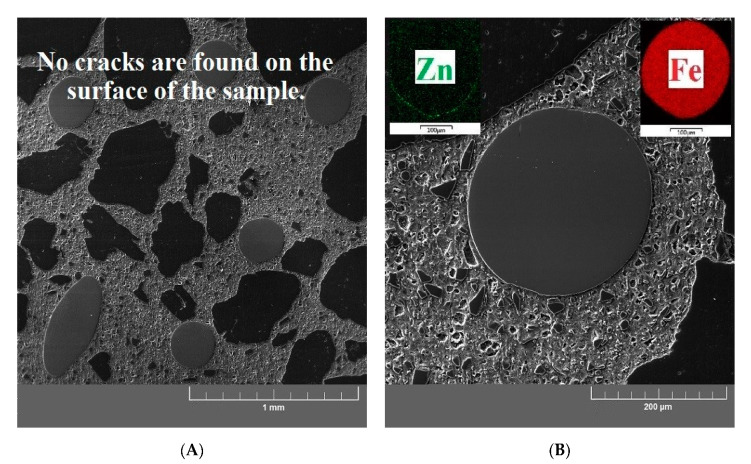
(**A**) SEM image of the reference sample focused on the UHPC matrix/aggregate interfacial transition zone. (**B**) SEM image of the reference sample focused on the brass-coated steel fiber/UHPC matrix interfacial transition zone.

**Figure 16:**
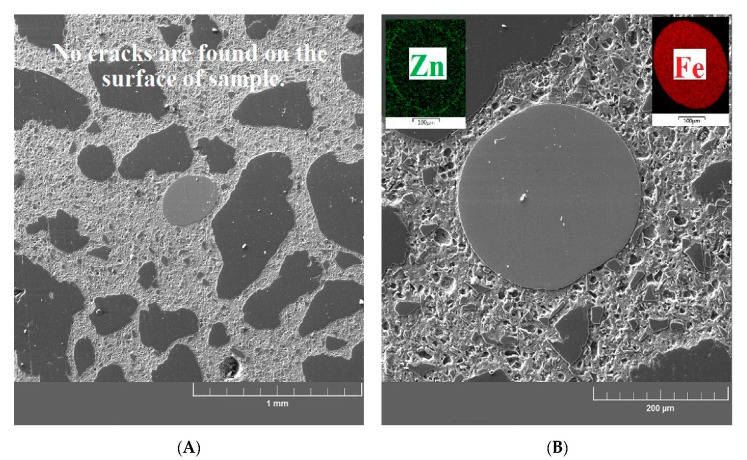
(**A**) SEM image of a sample after exposure to 200 °C focused on the UHPC matrix/aggregate interfacial transition zone. (**B**) SEM image of a sample after exposure to 200 °C focused on the brass-coated steel fiber/UHPC matrix interfacial transition zone.

Figure 15A,B show the cross sections of the reference UHPC specimen (exposure to 20 °C). These samples show a significant matrix integrity of the UHPC specimen. There are no apparent cracks, and the structure at the interfacial transition zone between the matrix/aggregate and the matrix/brass-coated steel fibers is compact. An identical structure without cracks in the matrix can be seen in the UHPC samples exposed to 200 °C (Figure 16A,B). An increased activity of silica fume caused by elevated temperature (pozzolanic reaction) was also confirmed in this experiment, manifested by the formation of dense micromorphology. General experience suggests that radial cracks can form in the cement paste at temperatures up to 100 °C. Cracks of this shape can arise when the thermal expansion coefficient of the paste is smaller than that of the aggregate (up to 100 °C, sand has usually a lower linear thermal expansion coefficient than the cement paste) [68]. Radial cracks were not observed in the UHPC matrix by SEM. Their possible local presence does not have a significant effect on the measured values of compressive strength (Figure 9). It can also be considered that they can be filled with cement hydration products due to the promotion of hydration process by elevated temperature.

**Figure 17:**
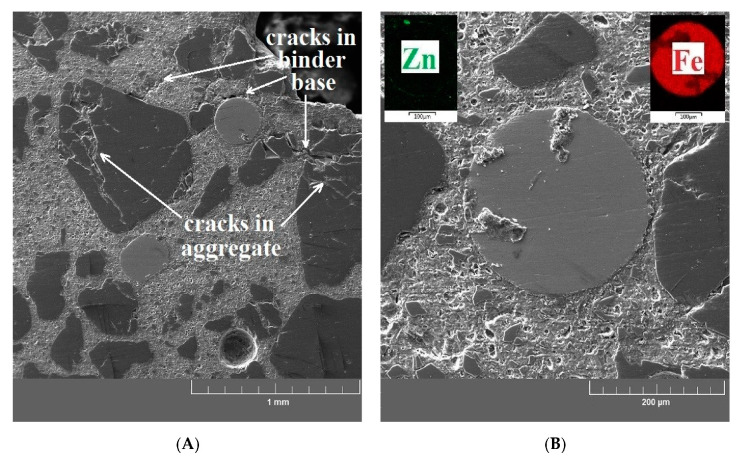
(**A**) SEM image of a sample after exposure to 400 °C focused on the UHPC matrix/aggregate interfacial transition zone. (**B**). SEM image of a sample after exposure to 400 °C focused on the brass-coated steel fiber/UHPC matrix interfacial transition zone.

**Figure 18:**
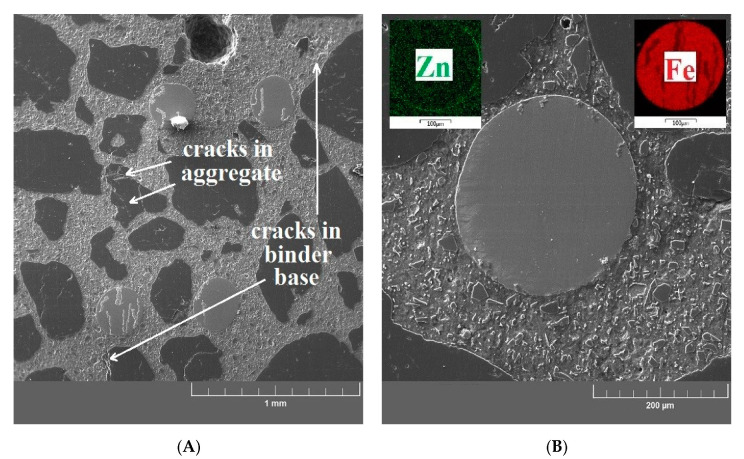
(**A**) SEM image of a sample after exposure to 600 °C focused on the UHPC matrix/aggregate interfacial transition zone. (**B**) SEM image of a sample after exposure to 600 °C focused on the brass-coated steel fiber/UHPC matrix interfacial transition zone.

**Figure 19:**
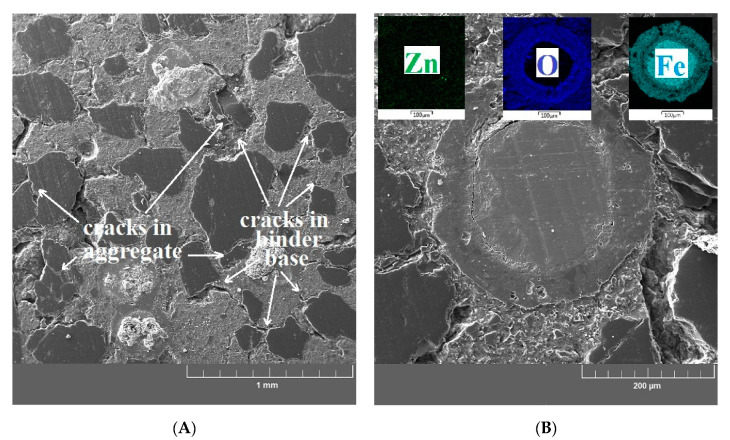
(**A**) SEM image of a sample after exposure to at 800 °C focused on UHPC matrix/aggregate interfacial transition zone. (**B**) SEM image of a sample after exposure to 800 °C focused on the brass-coated steel fiber/UHPC matrix interfacial transition zone.

A decrease of compressive strength and bond strength (UHPC with prestressing reinforcement) is apparent for the UHPC samples exposed to 400 °C. Considering the decrease of the mechanical properties of UHPC, this corresponds to the micromorphology of the UHPC matrix (see Figure 17A,B). For this temperature exposure, thin cracks occur in the binder base (mild deterioration) and in the aggregate. The cracks in the binder base (at the ITZ between the cement paste/aggregate) have a tangential shape and arise when the thermal expansion coefficient of the paste exceeds that of the aggregate. Inclusion cracks (cracks across the grain of the aggregate) arise in the same way [69,70,71]. Fine cracks were also detected at the matrix/aggregate interfacial transition zone; however, no cracks or other defects were found at the coated fiber/matrix interfacial transition zone. The surface of the brass-coated fibers is stable at this exposure temperature. The formation of cracks is caused by accumulated thermal stress, vapor pressure and, inconsistent expansion of the matrix and aggregate [14,72,73,74]. Significant cracks are apparent on the images of cross sections from the UHPC samples exposed to 600 °C (Figure 18A,B) and 800 °C (Figure 19A,B). In addition to the thermal stress, vapor pressure, and incompatible expansion, the formation of cracks at this temperature is also strongly affected by CH decomposition (Ca(OH)_2_-portlandite), CaCO_3_ (calcite), and C-S-H gel (see “X-ray powder diffraction”). This also corresponds to the increase of the total porosity of the UHPC specimen (see “Image analysis of confocal microscopy”) [7,75,76,77]. A significant portion of the cracks in aggregate (silica sand) is, above 573 °C, caused by the allotropic transformation of SiO_2_ from α-quartz to β-quartz with the volume expansion (~6%) [7,14,78]. A rapid decrease of compressive strength and bond strength is apparent for UHPC specimens exposed to temperatures between 400 and 600 °C. This can be attributed to the changes in the microstructure.

A smaller, however, still apparent decrease of the compressive strength and bond strength for the samples exposed to 800 °C is not related only to the propagation of the phenomena described above (due to increasing temperature), but also to deterioration and cracks at the matrix/brass-coated steel fiber phase interfacial transition zone. For the samples exposed to 800 °C, the melting effect for the brass coating is apparent and it is also visible that there is a significant reduction of the diameter of the coated steel fibers. Since the protective brass coating is very thin, it is dissolved even at 800 °C [79,80]. The diameter reduction due to surface oxidation, i.e., a partial melting of steel fibers, at 750 °C was described in [14,81,82] with a simultaneous reduction of hardness [83,84]. Crack formation on the phase interfacial transition zone of the brass coated fiber/matrix is predominantly caused by the variable thermal expansion coefficient of the matrix and steel fibers [80,81]. For the samples exposed to 800 °C the portion of cracks at the phase interfacial transition zone is filled by the products of thermal oxidation of the coated steel fiber (Figure 19B). However, a positive effect of this phenomenon on the compressive strength was not proven in this work. This finding is also in line with other studies on the topic [14,81].

## 4. Conclusions

This study investigates the changes of the bond strength between prestressing reinforcement and UHPC with elevated temperature. The bond of prestressing reinforcement in UHPC is tested for non-prestressed wires according to RILEM RPC 6 and CSN 73 1333 (pull-out tests). This procedure is adapted here for bond tests at elevated temperatures. Along with bond strength, the effect of elevated temperature on compressive strength, phase composition (detected by X-ray powder diffraction), and the microstructure of UHPC (mercury intrusion porosimetry, observation of SEM and confocal microscopy) is monitored.

Based on the extensive experimental campaign, the following conclusions can be drawn:

- Increasing the temperature up to 200 °C does not reduce significantly either the bond strength between prestressing reinforcement and UHPC, or the compressive strength of UHPC. This is why both variables, in this range of temperatures, are positively affected by the thermal increase of the reactivity of silica fume in the UHPC mixture.
- However, the bond and compressive strengths notably decrease with increasing temperature above 200 °C. As the presented results indicate that the rate of decrease is similar for both the bond and compressive strengths, it might be possible to estimate the decrease in bond on the basis of the observed decrease of compressive strength.
- More specifically, for temperatures between 200 and 800 °C, the bond strength is reduced by 20% (400 °C), 45% (600 °C), and up to 70% at 800 °C.
- The main factor leading to the observed reduction of the bond strength is the increasing porosity of UHPC—a small increase of porosity (e.g., the increase of 0.5% at 400 °C compared to the reference temperature) leads to a significant decrease of the bond (20%).
- The most significant increase of porosity is recorded between 400 °C and 600 °C (almost 2%), which is attributed to the formation of pores with a larger diameter.
- Changes of the phase composition of UHPC have a significant effect on the porosity only at higher temperatures (>600 °C).
- Exposure to 800 °C leads to the highest porosity increase, which is also caused by cracking in the UHPC matrix and the deterioration of the brass-coated fibers.

A future research should investigate the effect of relaxation of prestressing reinforcement with increasing temperature on the bond strength reduction by numerical modelling.

## Figures and Tables

**Table 1:** Cement composition guaranteed by producer (CEM II/A-S 52.5 N).

Compound	CaO	SiO_2_	Al_2_O_3_	Fe_2_O_3_	SO_3_	MgO	Na_2_O	K_2_O
Content, %	58.40	22.50	5.30	2.90	2.80	2.40	0.20	0.73

**Table 2:** Content of UHPC admixtures.

Admixture	Content (kg/m^3^)	Note
cement (CEM II/A-S 52.5 N)	700	cement composition—see Table 1
quartz sand	1275	maximal grain size guaranteed to 2 mm
silica fume	100	-
furnace slag	80	-
plasticizer (PCE HRWRA)	40	polycarboxylate base
brass coated steel fibres	120	1.5 vol.%; aspect ratio: 62.5
mixture (*w*/*c*)	0.25	

**Table 3:** Material characterization of prestressing reinforcement steel, parameters guaranteed by producer.

Property	Value and Unit
Ultimate tensile strength	1860 MPa
Nominal diameter	15.7 mm
Nominal density	~1172 g/m
Yield strength (0.1%)	246 kN
Young modulus	~195 GPa
Maximum elongation	3.5%
